# Cyber Teaming and Role Specialization in a Cyber Security Defense Competition

**DOI:** 10.3389/fpsyg.2018.02133

**Published:** 2018-11-19

**Authors:** Norbou Buchler, Claire Genevieve La Fleur, Blaine Hoffman, Prashanth Rajivan, Laura Marusich, Lewis Lightner

**Affiliations:** ^1^U.S. Army Research Laboratory, Adelphi, MD, United States; ^2^Industrial & Systems Engineering, University of Washington, Seattle, WA, United States; ^3^National CyberWatch Center, Largo, MD, United States

**Keywords:** cybersecurity, computer personnel selection, skill composition, expertise, teamwork, team development, collaboration, cyber defense

## Abstract

A critical requirement for developing a cyber capable workforce is to understand how to challenge, assess, and rapidly develop human cyber skill-sets in realistic cyber operational environments. Fortunately, cyber team competitions make use of simulated operational environments with scoring criteria of task performance that objectively define overall team effectiveness, thus providing the means and context for observation and analysis of cyber teaming. Such competitions allow researchers to address the key determinants that make a cyber defense team more or less effective in responding to and mitigating cyber attacks. For this purpose, we analyzed data collected at the 12th annual Mid-Atlantic Collegiate Cyber Defense Competition (MACCDC, http://www.maccdc.org), where eight teams were evaluated along four independent scoring dimensions: maintaining services, incident response, scenario injects, and thwarting adversarial activities. Data collected from the 13-point OAT (Observational Assessment of Teamwork) instrument by embedded observers and a cyber teamwork survey completed by all participants were used to assess teamwork and leadership behaviors and team composition and work processes, respectively. The scores from the competition were used as an outcome measure in our analysis to extract key features of team process, structure, leadership, and skill-sets in relation to effective cyber defense. We used Bayesian regression to relate scored performance during the competition to team skill composition, team experience level, and an observational construct of team collaboration. Our results indicate that effective collaboration, experience, and functional role-specialization within the teams are important factors that determine the success of these teams in the competition and are important observational predictors of the timely detection and effective mitigation of ongoing cyber attacks. These results support theories of team maturation and the development of functional team cognition applied to mastering cybersecurity.

## 1. Human dimension of cybersecurity

Despite the digital and virtual nature of the cyber domain, the dynamics of cyberspace are fundamentally human and adversarial. Broadly defined, the human dimension of cybersecurity involves the dynamic interaction of *attackers, defenders*, and *users*. Users pursue their defined goals (work and personal) that often require interacting with others and online systems using networked technology. Attackers seek to exploit both networked system vulnerabilities and increasingly the user community with social engineering attacks, whereas defenders monitor systems and attempt to thwart and mitigate any actions taken to compromise them. Most studies have focused on the vulnerabilities posed by the user, for instance to maintain compliance with security policies (Fulford and Doherty, 2003; Besnard and Arief, 2004; Werlinger et al., 2009) or in identifying insider threats (see Bishop et al., 2014; Costa et al., 2016). As a result, it is tempting to deride the human dimension as the weakest link in cybersecurity. However, humans are also the most adaptive and capable with respect to anticipating, reasoning about, and orchestrating an effective response and strategy to ongoing threats. There are many documented cases of the human contribution to reliability and resilience of complex, safety-critical systems (see Reason, 2017). Human defenders and analysts are also crucial to developing proper situational awareness and executing effective strategy. Cyber defense analysts do not work in isolation but as part of a cybersecurity team, and mastering cyber operations requires understanding what constitutes effective cybersecurity teaming.

Within the cybersecurity domain, examining effective teaming among cyber analysis involves understanding specific compositions of skills and roles among team-members as well as team-processes such as collaborative interactions and leadership. Initial research conducted at cyber defense exercises establishes methodological and analytical approaches (Malviya et al., 2011; Jariwala et al., 2012; Ogee et al., 2015; Granasen and Andersson, 2016; Henshel et al., 2016; Buchler et al., 2018) that have proven effective in such field work settings. Cyber defense exercises make use of simulation environments that provide some degree of experimental control and, critically, outcome measures of scored performance that objectively define overall team effectiveness. Since 2001, a wide variety of annual competitions and cyber defense exercises have emerged to support collective training and maturation of cyber defense teams. Collaborative defense is achieved through operational cooperation of different actors against common cyber threats and events (Klimburg, 2012). Such exercises provide a good opportunity to conduct field-based experiments on teamwork in cyber defense. Multiple teams comprising of security students or cyber defense professionals participate to perform live cyber defense tasks. Typically, a team captain of each participating “blue” team is identified as the leader and primary liaison. Examples include capture the flag style contests (Sharma and Sefchek, 2007; DEFCON, 2016), cyber defense competitions for high-school (Chapman et al., 2014), collegiate (Buchler et al., 2018) and professional levels (SANS Institute, 2016), as well as NATO and U.S. Military Cyber Defense Exercises (Ogee et al., 2015; Buchler et al., 2016a; Henshel et al., 2016). Cyber competitions emphasize a team approach in providing hands-on learning experience in the application of information assurance skills. The team competition is driven by a scenario that combines legal, ethical, forensic, and technical components in safeguarding the operation of critical information and its supporting infrastructure (Hoffman et al., 2005). In addition to demanding proficiency in cybersecurity skills, the competitions are explicitly designed to foster teamwork. Effective communication, collaboration, and leadership are necessary to manage the demands of applying practical information security skills in a live fire scenario with intense time pressure to perform against the clock. As scored competitions with clear metrics of evaluation, such events offer a unique opportunity for researchers to assess the contribution of various elicited cyber defense factors and explain their effectiveness against cyber attacks.

The overall goal is determining how the best, high-performing teams respond to and mitigate cyber attacks. Current assessments of how cybersecurity teams coordinate and work together to mount and conduct effective cyber defense operation relies on embedded observers. Their observational measures are essential to determine how the best, high-performing teams conduct cyber operations. Our approach builds upon prior work conducted at the preceeding yearly competition (Buchler et al., 2018) using factor analysis to examine observational constructs of collaboration and leadership style. For this year's competition, we also applied a new survey instrument to answer specific questions about team development such as the composition of cyber skills on each team and their level of expertise and experience. to examine factors contributing to effective teamwork and leadership in a cyber defense competition using observational (i.e., test and measures) and survey-based methodologies; a comparative analysis between both events is provided in the discussion. As a scored competition, this provides an explicit metric for team performance with which to compare team process, structure, skills, experience level, and leadership factors contributing to an effective cyber defense. The goal is to relate scored team performance during the competition to team experience, skill composition, and observational constructs of team collaboration and leadership.

## 2. Cyber defense skills

The cyber analyst work domain involves tasks and responsibilities for monitoring networks to detect suspicious and hostile activity that would jeopardize the integrity of information systems. To investigate known and potential indicators of network security breaches, cyber operators typically employ a number of security software tools, such as traffic monitors, firewalls, vulnerability scanners, and Intrusion Detection Systems (IDS). Defense analysts review logs from these various security tools and network traffic monitors in order to detect and then respond appropriately to anomalous network and system activity. This demands compiling information from various sources and preparing cybersecurity incident reports based on intrusions, events, and incidents that are detected and above any preset thresholds. A number of analytical procedures are typically employed as part of incident management. Recent research has begun to identify the requisite skills needed by team-members for effective team performance (Stevens-Adams et al., 2013). Pioneering work by D'Amico and colleagues (D'Amico et al., 2005; D'Amico and Whitley, 2008) examined individual cyber analyst workflows and identified several work categories for incident management, including the following: data triage analysis, correlation analysis, escalation analysis, threat analysis, forensic analysis, and incident reporting or response.

In general, data triage analysis is perhaps the most typical of these categories, involving handling and processing the large amounts of data generated by tools and monitors. Analysts must filter out false positives and prioritize efforts in line with their goals or mission. Correlation analysis ties together seemingly disparate events using current and historical data, connecting individual incidents uncovered by data triage. Escalation refers to when further investigation is needed, requiring greater situational awareness of the relevant environment and associated data, building on prior data triage and correlation. Forensic analysis focuses on gathering, securing, and preserving evidence of cyber attack or intrusion in a format that can be shared with and presented to law enforcement agencies and is admissable in a court of law. Proper forensic process enables analysis without alteration or tampering of source data. Incident reporting is often the primary outcome of defense analysts' work. Once the volume of data generated by detection tools and monitors is analyzed, any actual incident must be reported, creating a log of the detection supported with appropriate evidence and justification for the report. Incident reports also serve as a means to categorize and bin detections by severity and characteristics (e.g., attack type, affected machine(s), threat level). Lastly, threat analysis is a more pro-active analysis, using additional data sources such as news and information shared within hacking communities and the intelligence community to investigate potential attackers and attack strategies.

Each of these categories describes defensive activities that are centered on reaction, though threat analysis may enable proactive strategies. However, securing an asset within cyber operations entails more than comprehending alerts and logs and reacting to exploitation and intrusion. For example, even before an analyst can conduct a triage analysis some decision was made about which tools to install on a network to generate the alert data. As new information arrives and new technologies emerge, new defensive techniques and methods are developed and may be put in place, and there is then a need to validate and test these to confirm functionality and success. Detection and reporting alone do not fix exploited systems or intrusions, so operations must also patch vulnerabilities and address known exploits. A networked organization has various assets and services that must remain operational in the conduct of work despite potential intrusions or exploitation. This necessitates defensive operations that minimize impact and support uptime of work-relevant systems. Cyber defense benefits from an integration of cyber skill-sets that include technical, social, and strategic components. Cyber teaming emphasizes the need for multiple roles and skill-sets among team members.

The cyber domain includes both human and technical aspects and is heavily reliant upon the decision-making capabilities and skill-sets of defenders to overcome attackers and protect end-users. Each year, in addition to the many news reports of cybersecurity incidents and breaches, there are an increasingly number of security reports published by industry professionals. For instance, the 2017 Data Breach Investigations Report compiled by Verizon, details the methods, motivations, and targets of cyber crime for the prior year. The collection of analyses shows that hacking, malware, and social engineering remain the top three means of a data breach (Verizon, 2017). The report provides a standard Vocabulary for Event Recording and Information Sharing (VERIS), defining cyber incident categories: (i) hacking is the attempt to intentionally access or harm an information asset without or exceeding given authorization, (ii) malware is malicious software (scripts, code) run on a device to alter its intended function without consent, and (iii) social engineering is the use of deception and manipulation on human elements—often users—of information assets. Recommendations throughout the report encourage keeping up-to-date patches and software as well as thorough testing of defenses (Verizon, 2017).

Across the cyber domain, putting a tool or technique into action is often the only or best means of testing capabilities. In other words, system defenses are validated by attacking it. Red teaming involves taking on the mantle of an attacker using authorized attacks on a network, system, or tool to conduct evaluations of defensive effectiveness, functionality, and relevance to critical security goals. Red teaming and subsequent target assessments provide insights to improve operational procedures (Dunlap, 1998) as well as the validation and verification of tools and techniques and the development of novel approaches (Mirkovic et al., 2008; Rajendran, Jyothi and Karri, 2011). Cyber competitions use red teaming to evaluate defense team effectiveness, these include capture the flag (CTF) or national team competitions. In competitive settings, a whole spectrum of skills relevant to cyber operations are necessary and put to the test, including both technical and soft teamwork skills (NICE, 2017). How defenders collaborate, organize, and analyze problems is just as important as their technical acumen on the keyboard.

## 3. Cyber defense teaming

In defining the essence of professional teamwork, Hackman (Hackman and Katz, 2010) stated that teams function as *purposive social systems*, defined as people who are readily identifiable to each other by role and position working interdependently to accomplish one or more collective objectives. The responsibility for performing the various tasks and sub-tasks necessary to accomplish the team's goal is divided and parceled-out among the team. Team effectiveness often depends upon the appropriate leadership, skill composition, and necessary collaborations in the distribution of cognitive work.

Cyber defense teams battle with uncertain and unpredictable events in a networked operational environment. To address such challenges, analysts as a team, must be innovative, agile, and adaptable (Terreberry, 1968). Evidence from a handful of lab-based, empirical studies (Rajivan et al., 2013; Rajivan, 2014; Buchler et al., 2016b) highlight effective collaboration and leadership as critical determinants of performance for cyber defense given the complex and dynamic nature of the task domain. Managing the cognitive work of cybersecurity requires considerable interaction among teams of cyber analysts to monitor, report, and safeguard critical information technology. Communication is the key medium by which human teams form relationships, collaborate and share information. It is not imperative that all teams communicate extensively. The amount of communication necessary for effective performance differs based on team composition, type of task, and team maturity. Whatever the character of the team, however, some amount of effective communication is critical. Communication transforms individual knowledge and situational awareness to team level knowledge and situational awareness (Cooke et al., 2013). Such team level cognition emerges from effective team interactions. Effective team interaction (see Gist et al., 1987 for a review) is generally understood from an input-process-output framework focused on structural aspects (i.e., who talks to whom) and the team states that result in superior performance.

Examining teams in their natural work environment, as in cyber defense competitions, is essential to understanding how they work together to complete tasks and compete successfully. Few studies have addressed the specific composition of skills needed by cybersecurity teams. Efforts to characterize the specific composition of cybersecurity teams may be narrowly tied to the specific context and particular idiosyncrasies of a given cybersecurity exercise and scenario. Such conclusions might be relevant to practitioners of particular cyber security exercises but not to build general principles that can be applied across team contexts to multiple exercises. Our approach seeks to identify individual skills and competencies across team members that reflect the breadth and depth of capabilities expected of cyber defense teams and team processes generalizable to cyber operational settings. Such domain general approaches are needed in order to pave the way for more focused approaches using social-sensing technological platforms and big data analytical approaches that can be attuned to address the particular team context. Discovering the set of common tasks faced by cyber defense teams and their underlying decision-theoretic information and skill requirements is a particular area of emphasis.

## 4. Methods

### 4.1. Mid-atlantic collegiate cyber defense competition

**Figure 1:**
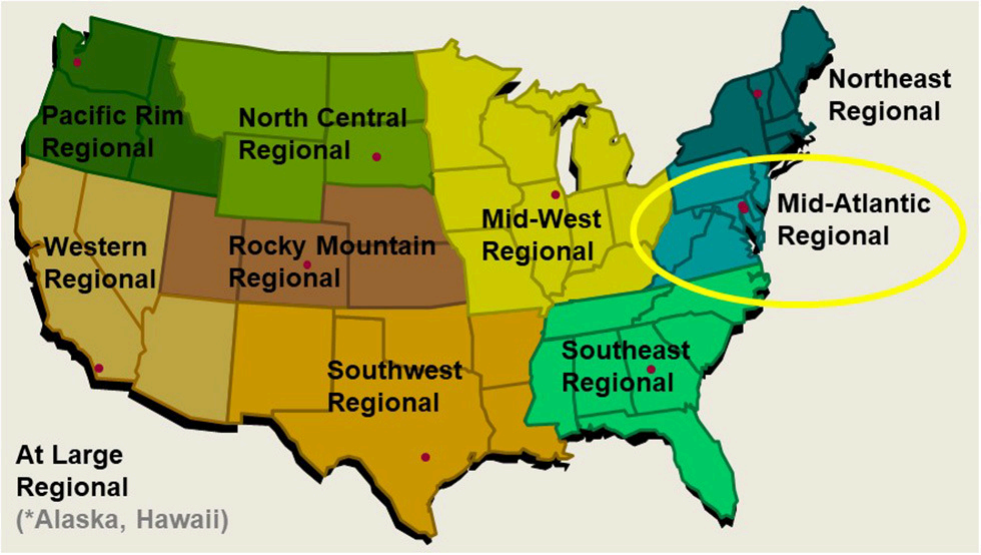
The 10-region National Collegiate Cyber Defense Competition with Mid-Atlantic Region circled.

The National Collegiate Cyber Defense Competition (NCCDC; www.nationalccdc.org) is an annual event involving thousands of students from hundres of colleges and universities across the United States, organized by the National Cyberwatch Center^1^ The NCCDC has three tiers of progression, starting at state or regional qualifiers. Successful teams proceed on to their region's competition, and the winners of each region move on to the final competition at the national level. Our study took place during the regional tier Mid-Atlantic Cyber Defense Competition (MACCDC), hosted at Johns Hopkins University Applied Physics Laboratory in Maryland (Figure 1). The MACCDC scenario differs from typical cyber competitions by incorporating real-world business activities and needs into defensive operations, covering routine business tasks as well as specialized server and network administration (White and Williams, 2005). Additional specifics regarding discussion of scenario development for the CCDC can be found in Mauer, Stackpole and Johnson (2012). Scenario tasks required teams to complete tasks common to an information technology department in a small- to medium-sized business rather than focus solely on cyber defense operations.

### 4.2. Man vs. machine scenario

**Figure 2:**
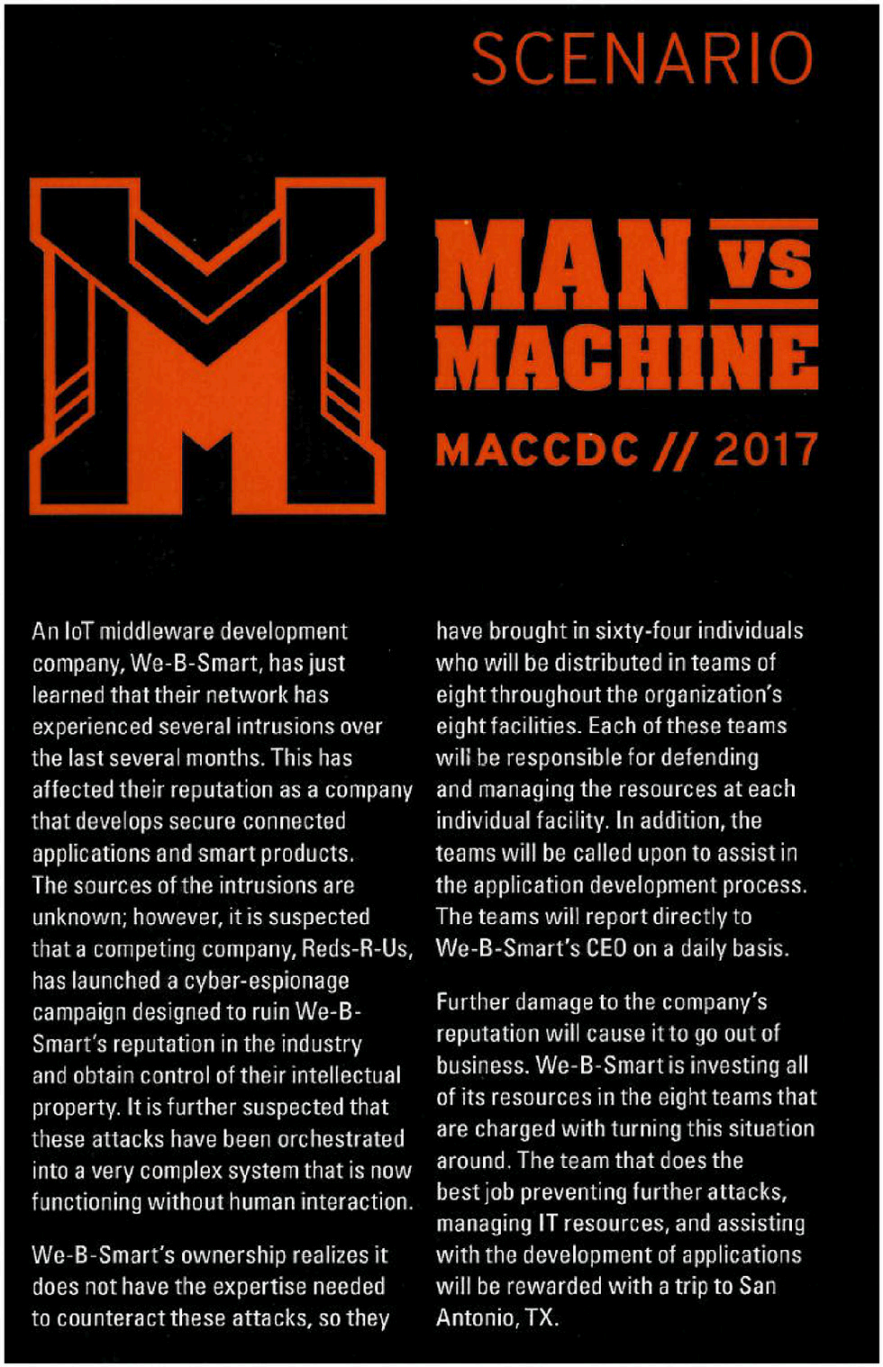
“Man vs. Machine” Internet-of-Things scenario description given to participants.

The scenario involved a cyber attack campaign with the intent to disrupt critical U.S. commercial infrastructure. The target was a fictional Internet of Things (IoT) middleware development company called We-B-Smart. The role of the participating MACCDC teams within the scenario was as additional cybersecurity brought in to work in concert with the respective information technology department of this commercial software firm. The eight teams were tasked in the 12th Annual MACCDC “Man vs. Machine” Scenario (see Figure 2) to initiate planning and to take over operations, cyberdefense support, and resource management for the targeted commercial facility. Each participating team (Blue Team) in the exercise consisted of 8 members. Each team was assigned a computer network and was asked to defend the network from simulated and real-time cyber attacks from an attacker team (Red Team) and handle requests and service needs (injects) during the exercise. Each Blue Team was identified using a unique team ID. Participating teams performed several defense activities over the course of 2 days during MACCDC. The White Team was responsible for generating network traffic that simulated day-to-day activities of an organization. One White Team member was assigned as an embedded observer to each team to closely monitor team activities and adherence to fair play competition and deliver inject events to team leads. During the exercise, the Red Team followed a scenario playbook of predefined goals over the timeline of the competition. Sample goals included compromising a server, stealing data, defacing websites, and modifying records.

### 4.3. Scored team performance

The importance of task type is well-established as an over-riding contextual variable in the organizational and teaming research literature (Beal et al., 2003), whether the focus is leadership style (Weed et al., 1976), group structure (Stewart and Barrick, 2000) or group coordination (Kabanoff and O'Brien, 1979). Consequently, many researchers examining group and team level processes have had to propose taxonomies of task-type (e.g., Hackman and Morris, 1975; McGrath, 1984). In our analysis of the cyber defense competition, a key advantage is that the task categories are provided by the event itself and are well-defined along the scoring dimensions. Ultimately, the MACCDC task categories were selected by the event coordinators at the National Cyber Watch Center (www.cyberwatch.org) as representative of the cyber defender workflows of an information technology department in a small- to mid-sized business.

**Figure 3:**
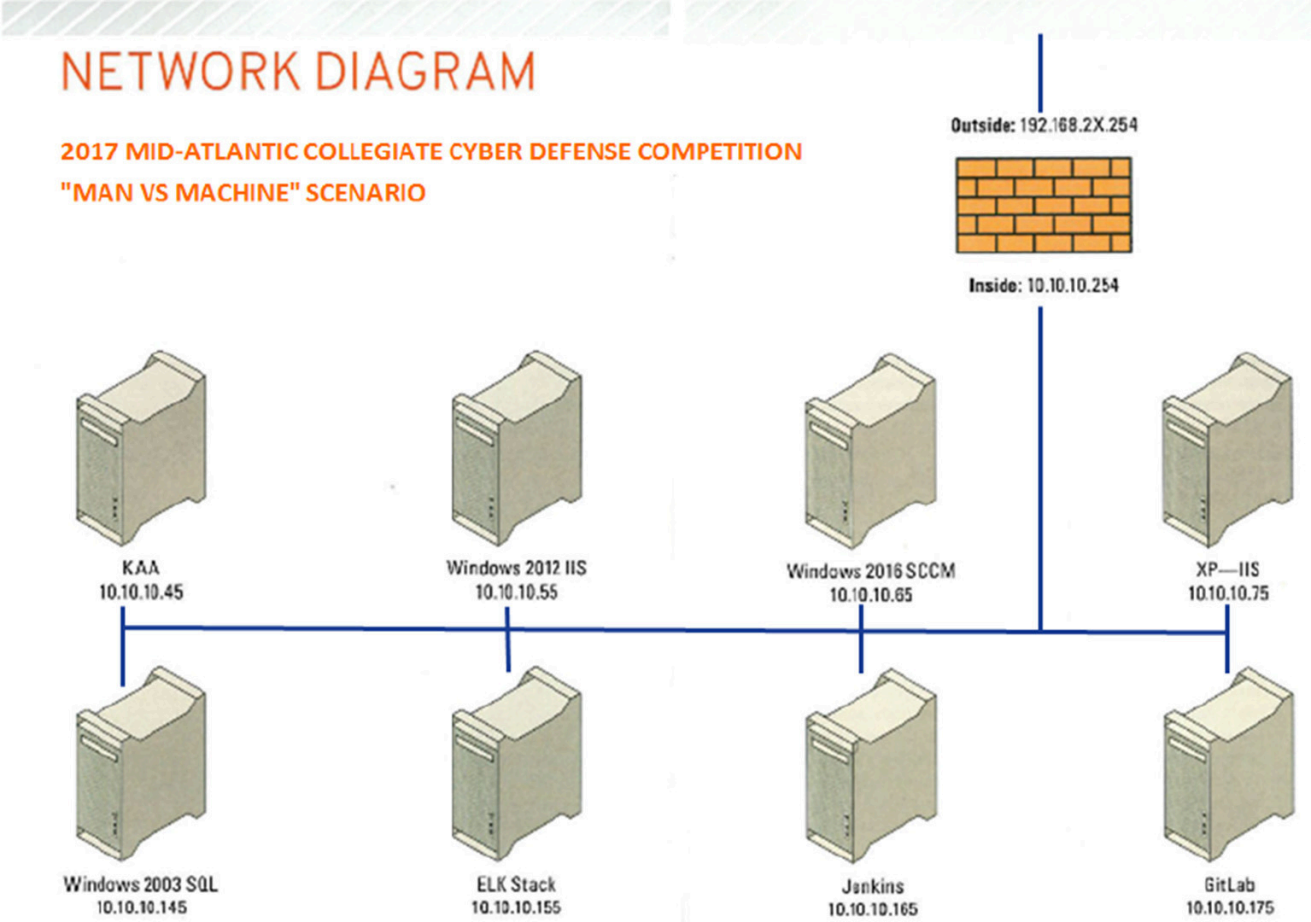
The network diagram of the We-B-Smart network.

**Figure 4:**
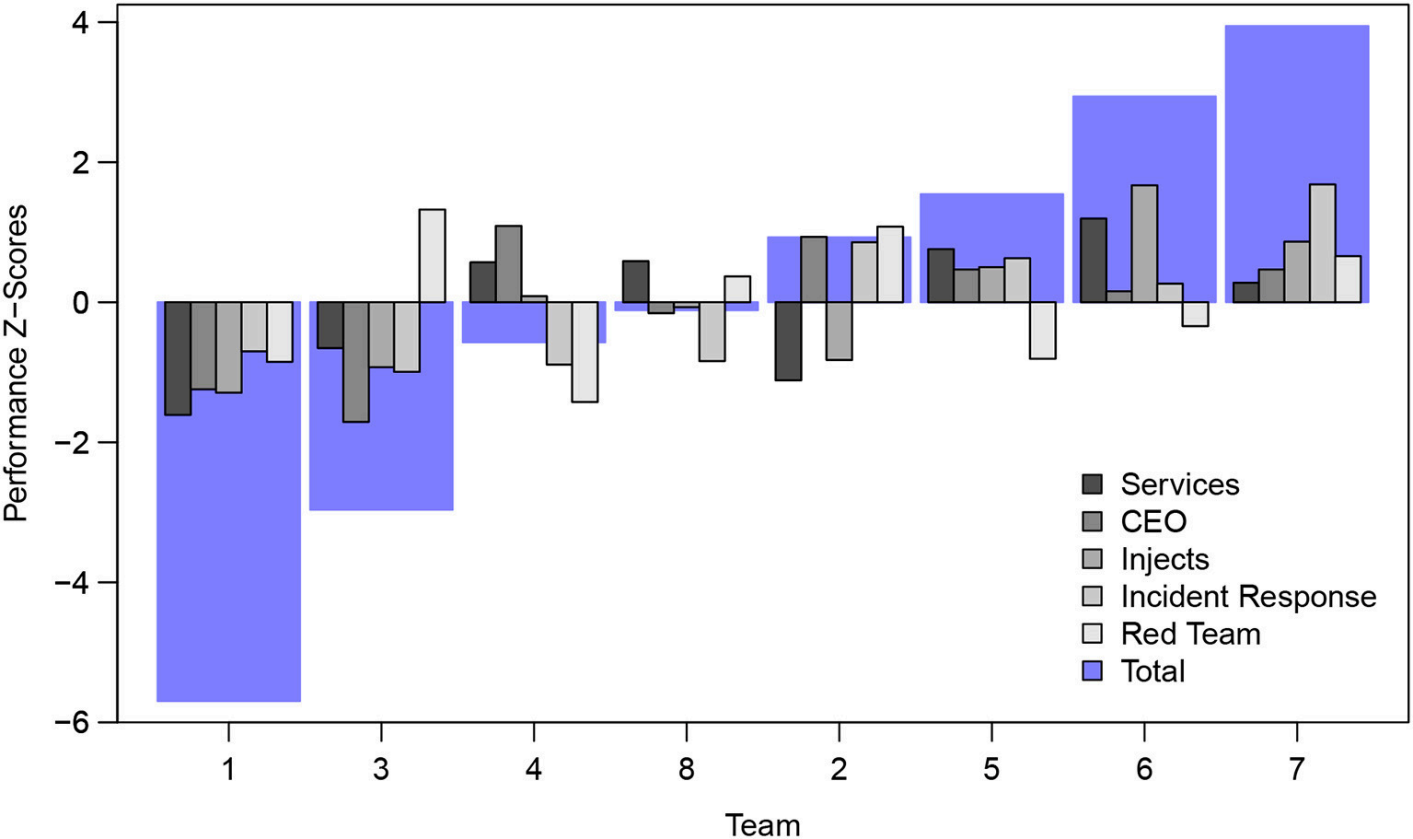
The composite overall team score in the MACCDC 2017 competition was composed of five performance metrics. These included: (1) Maintaining Services, (2) CEO Reporting, (3) Scenario Injects, (4) Incident Response, and (5) Red Team Activity. Performance scores are normalized z-scores and the teams arrayed with increasing composite performance by team number.

The teams were scored and ranked on five performance metrics over the course of the competition. Shown in **Figure 4**, the task-type categories included: (1) Services, (2) Scenario Injects, (3) CEO, (4) Incident Response, and (5) Red Team. First, all scored services had to be effectively managed by the teams to remain up and available with a high degree of integrity. Each service was given a predefined point value and checked using a custom Perl script running on a scoring server that automatically and periodically assessed network and service availability and integrity. Service scores were continually updated and displayed by the scoring server on a large screen by the teams' play area, providing viewers with real-time information about this performance category. The ten services that had to be maintained are shown in Figure 3—a MACCDC network diagram. To maintain the integrity of their network—in addition to keeping these services running and accessible—teams were required to defend competition “flags” within their network against theft by the adversarial Red Team. If the flag, a digital file, was captured from the Blue Team's environment or altered, the Blue Team would lose out on possible points earned. The more flag points a team defends against capture the better. Second, for the scenario injects, teams received tasks that needed to be complete within a given amount of time. The tasks were representative of a service delivery model for information technology departments of small- to mid-size businesses and included creating policy documents, making technical changes, and attending meetings. If the inject was completed on time and to the standard required, the team received the appropriate number of points. Third, each team designated a team leader who was periodically required to meet one-on-one with the Chief Executive Officer (CEO) of the commercial middleware company and respond to the demands of the Man vs. Machine scenario. The CEO ranked the team leaders based on their coherence and responsiveness in completing their assigned responsibilities. Teams were strongly encouraged to provide incident reports for each Red Team incident they detected. Incident reports were required to contain a description of what occurred (including source and destination IP addresses, timelines of activity, passwords cracked, access obtained, damage done, etc.), a discussion of what was affected, and a remediation plan. A thorough incident report that correctly identified and addresses attacker activity would potentially reduce the Red Team score (penalty) for that event or result in an “arrest” of the attacker—no partial points were awarded for incomplete or vague incident reports. Finally, the activities performed by the Red Team had a direct impact on the team score, emphasizing the need for Blue Teams to work to prevent Red Team activities. The Red Team had specific goals during the event, and each goal was assigned a point value. If the goal was accomplished, the Red Team was awarded the points and the Blue Team had a corresponding amount of points deducted from their score. For example, a Red Team player had the goal of obtaining a specific file off a Blue Team's mail server. The goal was worth 250 points. If the Red Team player acquired the file, they received 250 points, and the victim Blue Team had 250 points deducted from their score.

As shown in Figure 4, there was a good variation in composite team scores across all five metrics of performance. Our approach used observations and survey measures to distill key factors that may predict overall performance scores of the cyber security teams using survey instruments and structured observational approaches.

### 4.4. Procedure

This study was carried out in compliance of federal and Army Research Laboratory regulations requiring Institutional Review Board review of all research involving human subjects prior to the initiation of a research protocol to ensure the safe and ethical treatment of humans as subjects in research. All students were above the age of 18. The MACCDC event was conducted from March 31st to April 1st of 2017. During the orientation before the event on March 30th, researchers presented the study in a plenary session explaining the data collection effort to solicit voluntary participation by the students in the competition. They were informed that their teamwork would be observed and evaluated by an independent evaluator and that no video or audio data would be collected. They were also informed that no personally identifying information (PII) would be used in any of the evaluations. Since there were 64 participants, participant teams were briefed about the research project in a plenary session and acknowledged informed consent to participate in this research. Participant teams were not paid for their participation. They were thanked for their participation in the research.

Embedded observers were assigned to each participating Blue Team and closely monitored their activities. As part of the White cell overseeing the competition, they were not permitted to interact with the team directly. Embedded observers are typically used in cyber defense exercises to assess team performance and, in some cases, to collect team process measures (see Granasen and Andersson, 2016). We followed this best practice and had each embedded observer evaluate their Blue team daily using our Observational Assessment of Teamwork (OAT) scale over the course of the 2-day competition. The 13-item OAT scale is used to assess and evaluate the qualitative aspects of teamwork. Each item links to a different team behavior and process hypothesized to be relevant to cyber defense teams. Categorically, the 13-point OAT statements represent five dimensions of teamwork: (1) Task Distribution, (2) Team Discussions, (3) Leadership, (4) Communication, and (5) Collaboration. Each statement was scored on a 7-point Likert scale from 1 (strong disagreement) to 7 (strong agreement). At the start of each day of competition, all the embedded observers were debriefed on the specific teamwork processes, pertinent to OAT, they needed to observe during the course of the competition. They were asked to rate the teamwork behaviors of a team on an absolute scale and avoid comparing one team to another. At the conclusion of the competition, the Skill and Experience Survey was distributed to and completed by all participants.

## 5. Results

### 5.1. Skill and experience survey

Within the cybersecurity domain, examining effective teaming among cyber analysis involves understanding specific compositions of skills and roles among team-members. A skill and experience survey was administered to examine the underlying skill composition and experience level of cyber teams. Team members provided information about their experience-level and the set of cyber skills that they contributed to the team. Consistent with theoretical perspectives on team development (for a review, Kozlowski and Bell, 2003), we hypothesized that these real collegiate cyber defense teams undergo a maturation process over time by which they learn to work effectively together. Based on Tuckman's (1965) stage theory of team development and our sociometric results from the preceding 2016 MACCDC competition (Buchler et al., 2018), we hypothesized that more mature teams would develop functional role specialization and thus have a greater depth and breadth of skills. Furthermore, the cyber operational assessments conducted by Verizon (2017) suggest that the skills needed by a team should vary across threat categories and task domains.

#### 5.1.1. Team skill composition profile

To address whether more mature teams have different skill compositions than novice teams, we conducted a cluster analysis (using R and the complete linkage method). This cluster analysis examined the skill composition of teams as the percentage of team members endorsing each particular skill/role. Our inclusion criteria were skills or roles endorsed by at least 25% of the total sample. The cluster analysis sorted the teams into three main similarity clusters using a dendogram cut-point of 0.8 (see **Figure 7**); we labeled these team clusters as novice teams, proficient teams, and expert teams.

**Figure 5:**
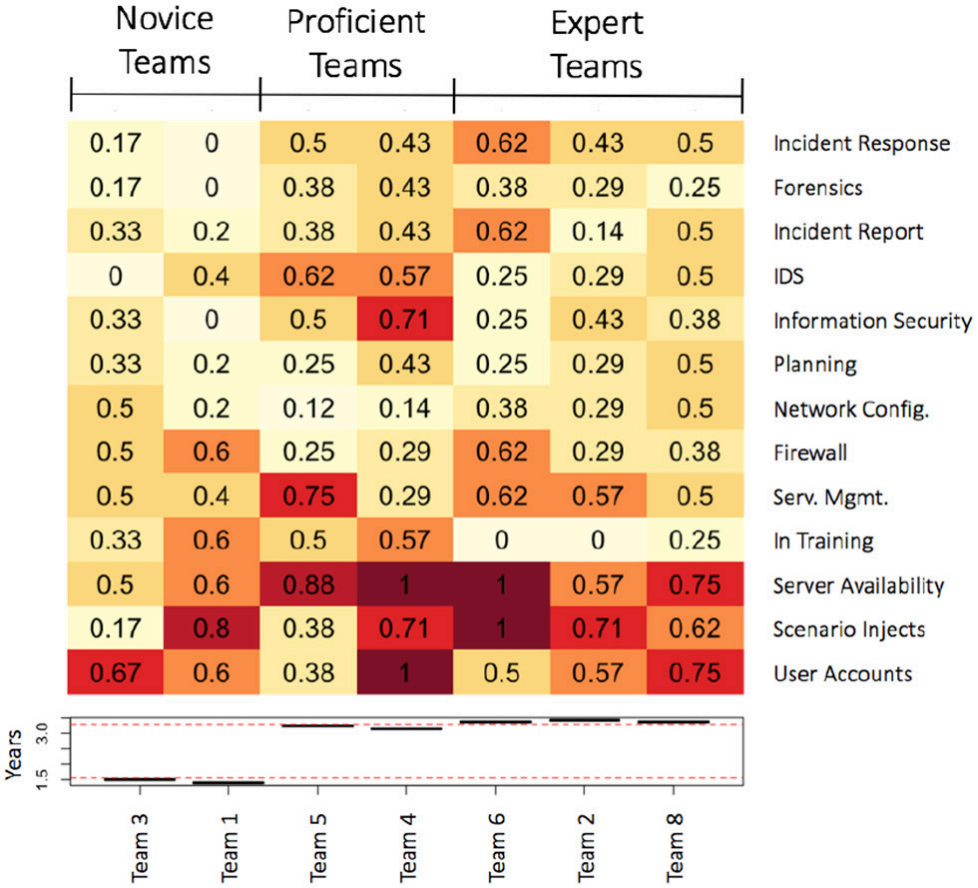
**(Top)** Heatmap representation depicting the skill composition of the various cybersecurity teams with darker colors indicating the proportion of team members endorsing a particular skill or role. **(Bottom)** Histogram of average years experience by each team.

Figure 5 represents these team skill composition profiles as a heatmap with darker colors indicating greater endorsement by a team of a particular skill or role. The Novice teams (first horizontal cluster) includes teams with the fewest years experience (Teams 3 and 1, *M* = 1.45 years). Looking at the proportion of skills represented in the Novice team cluster, it is evident that these teams lack both breadth and depth of skills. This is given by the sparse distribution of skills and roles endorsed and a substantial proportion (33 and 60%) of the team self-reporting as “in training” novices. The Proficient teams (second hortizontal cluster) includes two teams (Teams 5 and 4, *M* = 3.2 Years) with more experience than Novice teams but less than Expert teams. Most of the roles endorsed by Proficient teams endorsed at least 25% of the skills or roles. Half of the team members, however, self-reported as “in training,” suggesting that these teams have a breadth and depth of skills and knowledge that is not equitable. The Expert teams (third horizontal cluster) include three teams with the most experience (Teams 6, 2, and 8, *M* = 3.4 Years). Expert teams have a team skill composition profile similar to the Proficient teams cluster except there are few to no team members as “in training” novices; thus, the skilled expertise is broadly shared. Expert teams have both skill breadth and depth.

#### 5.1.2. Scored task domain analysis

The collegiate teams performed tasks common to an information technology department in a small to medium-sized business as the MACCDC emphasizes proficiency in task domains aligned with the service delivery model as practiced by information security professionals. These tasks included routine business tasks as well as specialized server and network administration functions (see Mauer, Stackpole and Johnson, 2012). To address how specific cyber skills and roles map onto the scored task domains, we conducted a second cluster analyses (using R and the complete linkage method). This cluster analysis examined the correlations between skill or role endorsement and team scored performance to determine essential skills needed for each task domain.

The analysis of the skills and roles yielded two vertical clusters (using a cutpoint of 1.0, see Figure S1 for a dendogram) that fall into two general categories System-level skills and Network-level skills. Network-level skills (first vertical cluster) require a broad view and knowledge pertaining to overall mission goals, such as the network topology, responding to the CEO's directives and needs, team leadership, and overall network policy and related rules. System-level skills (second vertical cluster) pertain to more detailed tasks and technical knowledge and activities needed to support and defend the network, such as parsing and evaluating individual alert logs, generating incident reports for an event from evidence, patching a vulnerability or misconfiguration, and managing a specific service and its associated settings.

**Figure 6:**
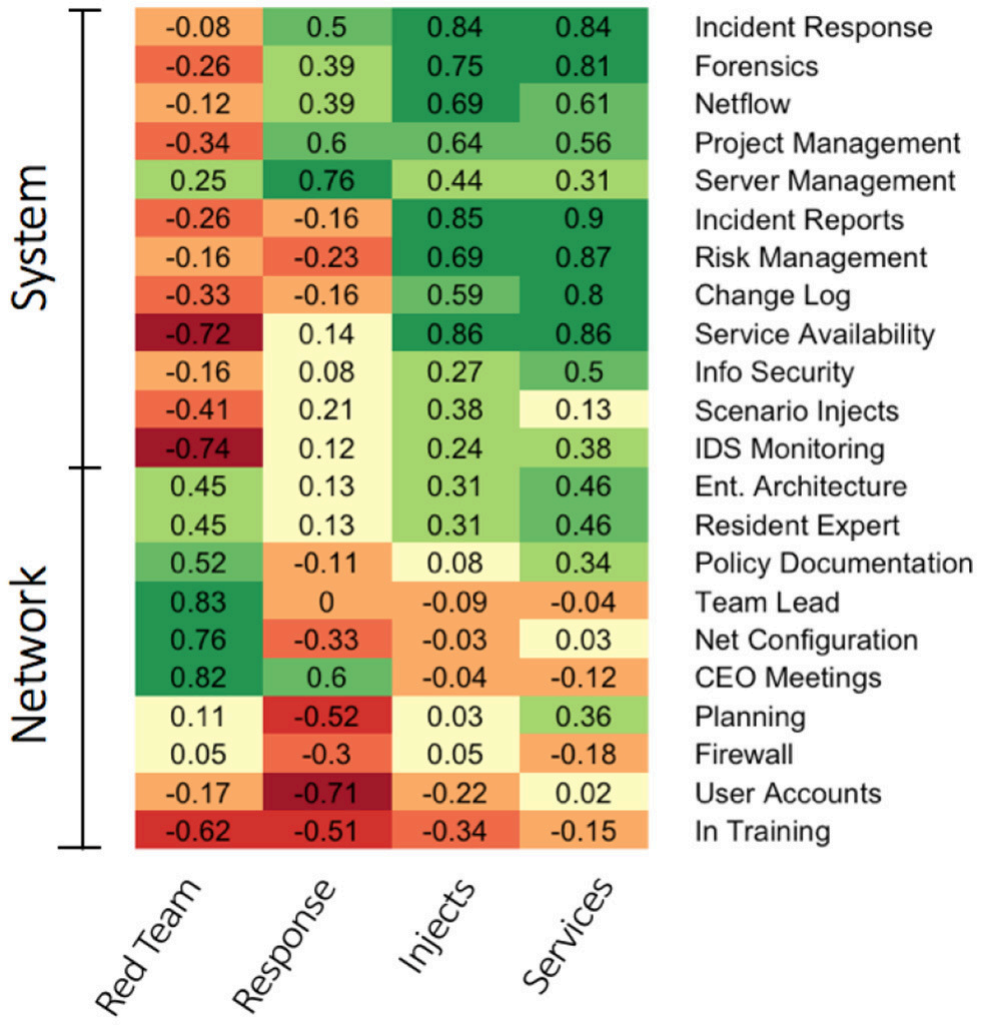
Heatmap representation depicting the skill composition of the various cybersecurity tasks.

Examining the scoring task domains, the analysis yielded three horizontal clusters (using a cutpoint of 1.0, see Figure S1 for a dendogram); each of the tasks were generally distinct except for (Scenario) Injects and (Maintaining) Services, which were clustered together. The task domains are represented in Figure 6 as a heatmap with darker greens signifying large positive correlations and darker reds signifying large negative correlations. Successful Red Team performance was positively associated with Network-level skills and negatively associated with System-level skills. Successful Incident Response performance was positively associated with System-level skills and negatively associated with Network-level skills. Finally, Scenario Injects and Maintaining Services were positively associated to System-level Tasks. In summary, as expected, an analysis of the cyber task domain scoring dimensions revealed that the MACCDC does indeed require a mix of cyber skills needed to address the diverse challenges presented by the event. Each scoring dimensions was associated with a diverse set of specific cyber skills, suggesting that a breadth and depth of skills are needed for successful outcomes at the competition. This finding aligns nicely with our previous result that Expert teams had a breadth and depth of skills.

### 5.2. Bayesian multiple linear regression

#### 5.2.1. Preliminary analysis

We employed a Bayesian analytic approach where observed data is used to produce complete distributional information regarding the parameters in a regression model (Buchler et al., 2018). We chose to use a non-committal broad prior on the parameters to ensure that the prior had minimal influence on the posterior, as we were testing new variables not included in our previous analysis. The data were standardized and the intercept and slope parameters had normal priors with mean zero and standard deviation of 10, which is very large relative to the scale of the standardized data (standardized regression coefficients will tend to fall between −1 and 1). The residual-noise parameter had a broad prior extending from zero to 10 (which is extremely broad and inclusive relative to the standardized noise of 1). The estimated parameters were linearly transformed back to the original scale (see Kruschke, 2011). The posterior was generated as a Markov Chain Monte Carlo (MCMC) sample using the R statistical computing software, rjags, and JAGS (Plummer, 2016). Three MCMC chains were initialized at the maximum likelihood values of the parameters and well burned in (for 1,000 steps), and a total of 250,000 steps were saved. There was very little auto-correlation in the well-mixed chains. The resulting MCMC sample is therefore highly representative of the underlying posterior distribution.

Four measures (Communication and Collaboration, Team Leadership, Years of Experience, and Number of Roles) were evaluated as predictors of four different performance metrics collected at MACCDC (Services, Scenario Injects, Red Team, and Incident Response). As several of our predictors were significantly correlated (see Table 1), we tested whether our data met the assumption of collinearity. We did this by calculating a Variance Inflation Factor (VIF), which measures how much variability of a coefficient is increased due to collinearity, for each of the predictors. When all four predictors were included, two of the predictors had VIFs over 5 (Communication and Collaboration = 14.74, Team Leadership = 21.08, Years = 2.67, Sum of Roles = 4.58). This suggests that multicollinearity was likely occurring (Kutner, Nachtsheim and Neter, 2004). However, when Team Leadership was excluded, VIFs for the remaining predictors indicated that multicollinearity was no longer a concern (Communication and Collaboration = 1.63, Years = 2.67, Sum of Roles = 3.15). Based on these findings, we conducted our regressions using these three predictors only. Separate regression models were used to predict the four different performance metrics.

**Table 1:** Correlations (and 95-percent Confidence Intervals) for the predictors.

	**1. Communication and collaboration**	**2. Leadership**	**3. Years of team experience**	**4. Number of skill roles**
1.	1	–	–	–
2.	0.95 (0.76, 0.99)***	1	–	–
3.	0.52 (−0.29, 0.90)	0.62 (−0.14, 0.92)	1	–
4.	0.61 (−0.15, 0.92)	0.75 (0.10, 0.95)^*^	0.79 (0.19, 0.96)*	1

* *p < 0.05*,

*** *p < 0.001*.

#### 5.2.2. Predict maintaining services score

We initially evaluated each of the measures as individual predictors of the Maintain Services score. The results of these analyses are presented in Table 2. For each measure, less than 10% of the 250,000 representative values in the posterior distribution were at or below zero for each of the predictors. Therefore, we can infer that greater communication and collaboration, more years experience, and a larger number of roles are all strong predictors of high scores for maintaining services.

**Table 2:** Means of posterior distribution (and 95% highest posterior density intervals) for each of the simple regression parameters.

	**Maintain-Services**	**Scenario-Injects**	**Red team**	**Incident-Response**
**COMMUNICATION AND COLLABORATION**				
r2	0.42 (−0.13, 1.00)	0.54 (−0.03, 1.13)	0.27 (−0.23, 0.79)	0.09 (−0.24, 0.41)
oz	1.07 (−0.48, 1.91)	0.95 (−0.42, 1.71)	1.21 (−0.54, 2.15)	1.34 (−0.59, 2.39)
β0	−0.00 (−0.82, 0.82)	0.00 (−0.71, 0.74)	−0.00 (−0.94, 0.90)^*^	−0.00 (−1.01, 1.03)
β1	0.65 (−0.21, 1.54)^*^	0.74 (−0.04, 1.53)^**^	−0.52 (−1.51, 0.45)	0.29 (−0.82, 1.38)
**YEARS OF EXPERIENCE COMPETING**				
r2	0.45 (−0.24, 1.14)	0.43 (−0.26, 1.10)	0.01 (−0.15, 0.18)	0.31 (−0.33, 0.95)
oz	1.14 (−0.44, 2.19)	1.14 (0.46, 2.19)	1.52 (−0.60, 2.91)	1.27 (−0.50, 2.44)
β0	−0.00 (−0.96, 0.95)	−0.00 (−0.96, 0.95)	−0.00 (−1.26, 1.27)	−0.00 (−1.07, 1.06)
β1	0.67 (−0.36, 1.69)^**^	0.66 (−0.39, 1.67)^*^	−0.12 (−1.47, 1.27)	0.56 (−0.60, 1.71)
**NUMBER OF ROLES**				
r2	0.90 (−0.49, 1.33)	0.66 (0.00, 1.31)	0.08 (−0.30, 0.46)	0.00 (−0.07, 0.08)
oz	0.48 (−0.19, 0.94)	0.89 (0.35, 1.71)	1.46 (−0.58, 2.81)	1.52 (0.61, 2.93)
β0	0.00 (−0.42, 0.40)	0.00 (−0.75, 0.74)	0.00 (−1.25, 1.20)	0.00 (−1.29, 1.26)
β1	0.95 (−0.52, 1.40)^***^	0.81 (−0.00, 1.61)^**^	−0.29 (−1.61, 1.02)	0.06 (−1.31, 1.42)

*() are 95% highest posterior densities*.

* *Less than 10% of b1 parameters smaller or equal to 0*.

** *Less than 5% of b1 parameters smaller or equal to 0*.

*** *Less than 1% of b1 parameters smaller or equal to 0*.

**Figure 7:**
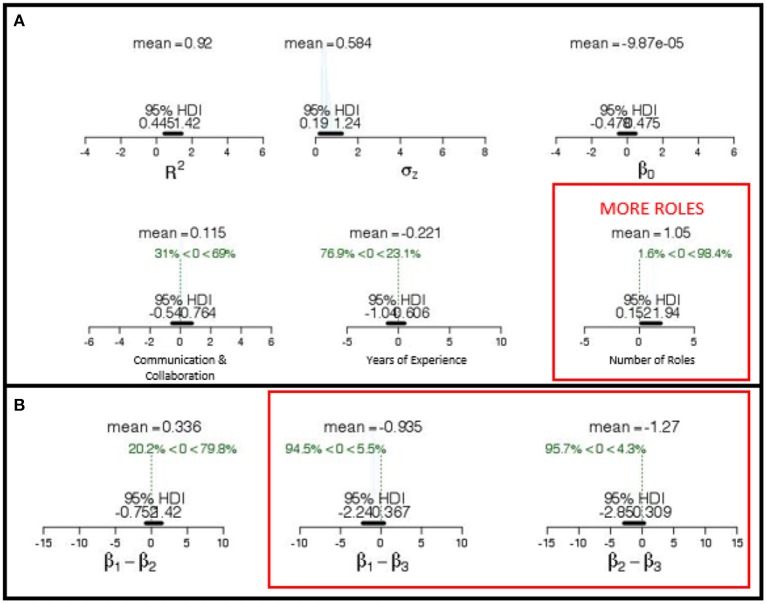
**(A)** Posterior distributions of regression parameters for Model 1 predicting Maintaining Services score with β*1* (Communication & Collaboration Factor), β*2* (Years Experience), and β*3* (Number of Roles) as predictors of team performance. Strong predictors (Number of Roles) indicated by red type. **(B)** Posterior distributions of difference among parameters indicate unique predictive quality of β*1*, β*2* and β*3* to Maintaining Services scored performance.

We then tested a multi-variate model that included the Communication and Collaboration factor, years of experience, and number of roles as *simultaneous* predictors of team performance on scored Maintain Services tasks using a Bayesian Multiple Linear Regression statistical model. The marginal posteriors for the three predictors are presented in Figure 7A. The black bar and values at the bottom of the x-axis denote the credible value ranges within the 95% HDI (Kruschke, 2015).

The marginal posterior for the Communication and Collaboration factor had a mean of 1.15 and a 95% HDI that extended from −0.54 to 0.76. Since more than 15% of the credible values were at or below zero for this measure, we can infer that Communication and Collaboration is not a unique predictor of Maintain Services performance. The marginal posterior for the Years of Experience factor had a mean of −0.22 and a 95% HDI that extended from −1.04 to 0.61. Since more than 15% of the credible values were at or below zero for this measure, we can infer that Communication and Collaboration is not a unique predictor of Maintain Service performance. The marginal posterior for number of roles had a mean of 1.03 and a 95% HDI that extended from −0.15 to 1.94. Since less than 5% of the credible values were at or below zero for number of roles, we can infer a greater breadth of skills is uniquely beneficial for Maintain Services.

We can also infer that number of roles differed strongly from the other predictions because less than 10% of the credible values were at or below zero when the model coefficients are expressed as difference scores between Years of Experience/Communication and Collaboration and Number of Roles (see Figure 7B).

In summary, these results show that although these three predictors jointly measure latent factors related to service scores, such as the maturation of team processes, only number of roles was uniquely related to Maintaining Services scores. One explanation for these findings is that to maintain services, a successful team needs to have a broad availability of requisite skills.

#### 5.2.3. Predict scenario injects score

As with Maintaining Services, we initially evaluated each of the measures as individual predictors of Scenario Injects score. The results of these analyses are presented in Table 2. For each measure, less than 10% of the 250,000 representative values in the posterior distribution were at or below zero for each of the predictors. Therefore, we can infer that greater communication and collaboration, more years experience, and a larger number of roles are strong predictors of better performance on Scenario Injects.

**Figure 8:**
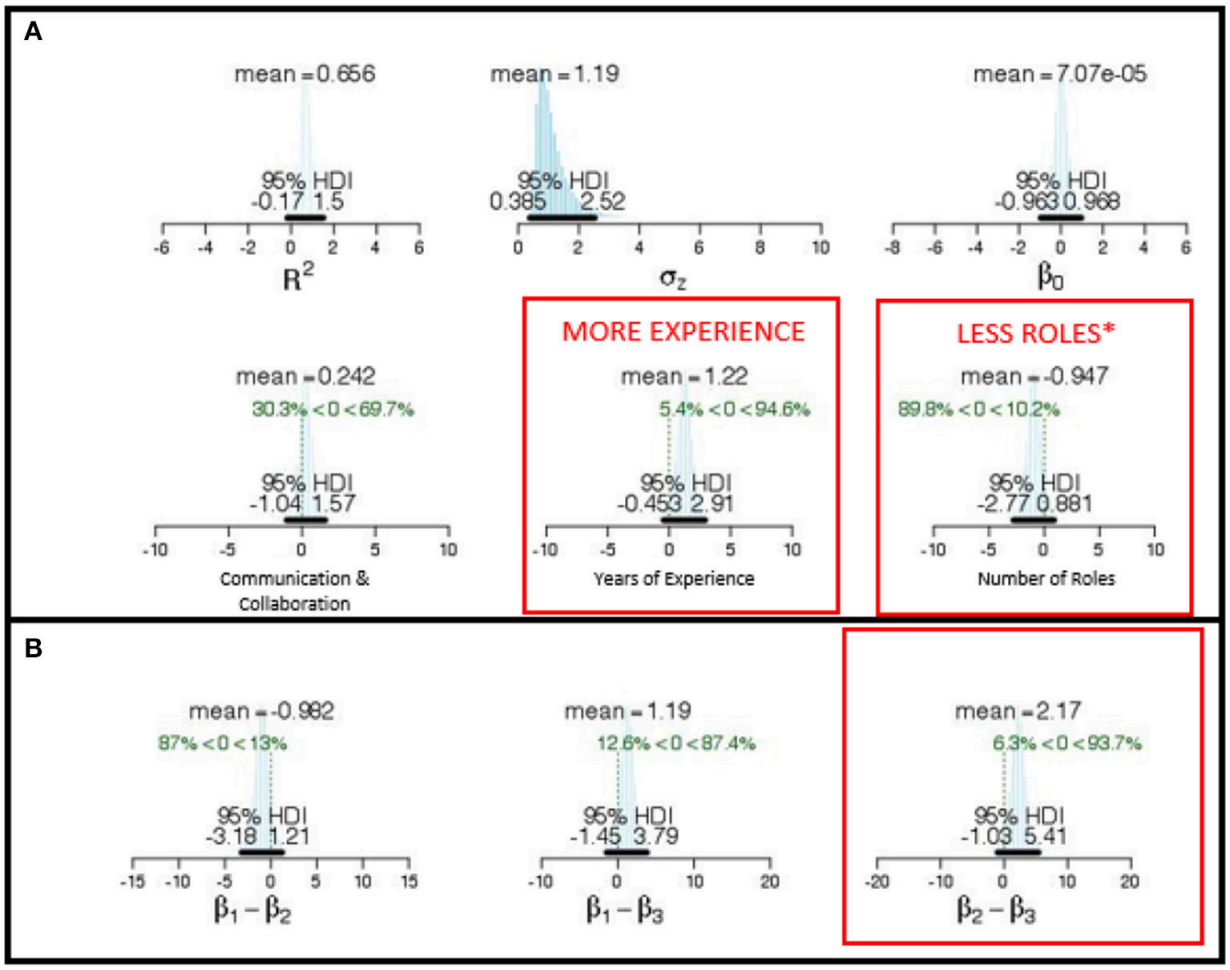
**(A)** Posterior distributions of regression parameters for the simultaneous model predicting Incident Response score with β*1* (Communication and Collaboration Factor), β*2* (Leadership Factor), and β*3* (Number of Skill Roles) as predictors of team performance. Strong predictors indicated by red type. **(B)** Posterior distributions of difference among parameters indicates unique predictive quality of β*1* and β*3* to Incident Response scores.

We then tested a multi-variate model that included the Communication and Collaboration factor, years of experience, and number of roles as simultaneous predictors of team performance on scored Inject tasks (see Figure 8B). The marginal posterior for the Communication and Collaboration factor had a mean of 0.37 and a 95% HDI that extended from −0.76 to 1.50. The marginal posterior for the Years of Experience factor had a mean of −0.11 and a 95% HDI that extended from −1.33 to 1.56. The marginal posterior for number of roles had a mean of 0.50 and a 95% HDI that extended from −1.04 to 2.09. Since more than 15% of the credible values were at or below zero for each of the three variables, we can infer that these measures are not uniquely predictive of Injects performance.

Similar to our previous multi-variate model, the communication and collaboration factor, years of experience, and number of roles appear to jointly measure the same latent characteristics, such as team maturation, that predict Scenario Inject performance. However, the factors are not uniquely predictive.

#### 5.2.4. Predict red team score

As previously, We initially evaluated communication and collaboration, years of experience, and number of roles as individual predictors of the Red Team score. The results of these analyses are presented in Table 2. For years of experience and number of roles, more than 15% of the 250,000 representative values in the posterior distribution were at or below zero for each of the predictors. Therefore, we can infer they are not strong predictors of better Red Team performance scores. For communication and collaboration, 11.9% of the variables were at or above zero, from which we can infer that less interaction between team members predict better performance against the red team.

We then tested a multi-variate model that included the Communication and Collaboration factor, years of experience, and number of roles as simultaneous predictors of team performance on scored Red Team tasks. The marginal posterior for the Communication and Collaboration factor had a mean of 0.63 and a 95% HDI that extended from −2.39 to 1.18. The marginal posterior for the Years of Experience factor had a mean of 0.46 and a 95% HDI that extended from -1.75 to 2.75. The marginal posterior for number of roles had a mean of −0.20 and a 95% HDI that extended from −2.67 to 2.16. Since more than 15% of the credible values were at or past zero for each of the three variables, we can infer that these measures are not uniquely predictive of Red Team performance. In general, our measures do appear to be relevant to Red Team performance.

#### 5.2.5. Predict incident response scores

As previously, we initially evaluated communication and collaboration, years of experience, and number of roles as individual predictors of the Incident Response score. The results of these analyses are presented in Table 2. For all three measures, more than 15% of the 250,000 representative values in the posterior distribution were at or below zero for each of the predictors. Therefore, we can infer they are not strong predictors of Incident Response scores.

We then tested a multi-variate model that included the Communication and Collaboration factor, years of experience, and number of roles as simultaneous predictors of team performance on scored Incident Response tasks (see Figure 8A). The marginal posterior for the Communication and Collaboration factor had a mean of .24 and a 95% HDI that extended from −1.04 to 1.57. Since more than 15% of the credible values were at or past zero for each of the three variables, we can infer that these measures were not uniquely predictive of Incident Response performance.The marginal posterior for years experience had a mean of 1.22 and a 95% HDI that extended from −0.45 to 2.91. Since less than 10% of the credible values were at or below zero, we can infer that having more years experience was a positive predictor of Incident Response.The marginal posterior for number of roles had a mean of −0.95 and a 95% HDI that extended from −2.77 to 0.88. Since only 10.2% of the credible values were at or below zero, we can infer that having fewer roles was a positive predictor of Incident Response. We can also infer that these two predictors differed strongly from one another because less than 10% of the credible values were at or below zero for the difference scores between them (see Figure 8B).

In summary, successful Incident Response performance appears to require a great deal of experience in competition as a team and team members with a smaller number of delineated roles. This suggests that skill depth is necessary to establish a winning incident response.

## 6. Discussion

At a premiere collegiate cyber defense competition, we conducted a series of analysis using derived measures from observational and survey-based instruments to predict team performance. As a well-established and moderated competition, the MACCDC provided a multi-dimensional evaluation of scored team performance along indices of: (a) maintaining services, (2) incident response, (3) scenario injects, and (4) handling red team attacks. Bayesian analysis predicted MACCDC team performance along each of these scoring dimensions using our derived measures of team processes. Our derived measures of team collaboration, team experience-level, and team skill-composition were validated as strong and unique predictors of scored team performance. An additional scoring dimension of red team defense was not predicted by our measures. These results are each discussed in relation to a theoretic perspective in the research literature on team development. We then integrate our current findings with the team effectiveness research literature and prior findings obtained from last years' MACCDC 2016 competition (Buchler et al., 2018).

### 6.1. Development of role specialization

We hypothesized that members of high performing teams would have functional role specialization, consistent with the Tuckman's (1965) stage model of team development. In this well-established ethnographic model, there are four stages to team development: (1) forming, where their focus is on understanding one another's skills and establishing shared approaches, (2) storming, where team members are in conflict, potentially over the control of team processes, (3) norming, where cooperative approaches are formed but have not yet been validated, and (4) performing, where the team has both defined roles for certain players and the flexibility to respond efficiently and effectively to the task at hand.

Consistent with our team composition hypothesis derived from Tuckman's model, clustering analyses revealed that low, middle, and high performing teams differed in composition. In the lowest performing “novice” groups, a number of the members were still in training and many of the roles were endorsed by few or none of the team members. These teams demonstrate a lack of depth and breadth of knowledge, consistent with the storming team development stage. In the middle “proficient” groups, most of the roles were covered by at least one person on the team but a large proportion of the team self-identified as *in training*. These “proficient” teams were consistent with the norming phase, where a team has some breadth of knowledge but lacks depth in technical skills. Finally, in the highest “expert” performing groups, few to no members were *in training* and all roles were assumed by at least one team member. These “expert” teams align with the performing stage, where teams have both the breadth (a wide range of roles/skills) and depth of knowledge (specialized knowledge in key areas) necessary to efficiently and effectively respond.

### 6.2. Role specialization by task

We were also interested in determining which particular skills contributed to performance in each of the scored task domains: Maintaining Services, Scenario Injects, Red Team, and Incident Response. We found that specific sets of cyber skills were associated with each of the four task domains. This is consistent with our previous findings that high performing expert teams were composed with a breadth and depth of skills. Below a cluster analyses is provided using the cyber work categories provided by D'Amico and Whitley (2008).

We found that cybersecurity skills fall into two general categories: System-level skills and Network-level skills. Network-level skills required a wide range of proficiencies that are dependent upon the security needs and protocols of the organization. These skills require ingenuity to anticipate potential problems and require the use of analytical approaches to fix problems and maintain system security. System-level skills required more detailed analysis of security risks and evaluation of specific systems and configurations, such as firewalls, intrusion detection systems, and interrogating network events and establishing security status.

The Maintaining Services score represents a team's ability to keep necessary systems accessible and operational despite potential intrusion and exploitation. In our task domain analysis, the skills that contributed most to high performance scores for Maintaining Services included System-level skills, such as incident reporting and response, service availability, and risk management. This characterization corresponds to data triage, correlation analysis, and appropriate escalation workflows described in prior cognitive work analyses (D'Amico). Not surprisingly, many of these skills also contributed to high Scenario Inject scores, where teams received a variety of high-priority tasks that needed to be completed within a given amount of time. These tasks were consistent with a service delivery model involving the creation of policy documents, system administration activities, and technical implementations.

Red Team score involves detection of malicious behaviors and actions to mitigate attacks on the network. This requires threat analysis in conjunction with data triage, correlation analysis, and proper incident reporting. In our task domain analysis, the skills that contributed most to high performance scores for thwarting red team actions included Network-level skills, such as network configuration but also high-level team management (team leadership) and understanding of adversarial intent (attending CEO meetings). In general, the skill composition of these high-performing teams did not include System-level skills. It may be, however, that these teams lacking technical System-level skills chose to defend their networks against red team actions. The strategies used by teams to allocate skills and resources to the various task domains is a topic of future inquiry.

Incident Response scores reflect the ability to respond to emerging threats and detected intrusions, relying on data triage analysis, forensic evidence as can be collected, and proper incident reporting. Teams were required to produce professional reports as no partial points were awarded for incomplete or value incident reports. This included a description of what occurred (including source and destination Internet Protocol addresses, timelines of activity, passwords cracked, access obtained, damage done, etc.), a discussion of what was affected, and a remediation plan. In our task domain analysis, the skills that contributed most to high performance scores for Incident Response included System-level skills such as incident response, server management, project management, netflow, and forensics.

### 6.3. Collaboration and years of experience

Our Collaboration factor—derived from the scaled observational assessment—was positively associated with the Scenario Inject and Maintaining Services scores. Yet, this factor did not independently predict scored performance when the other two measures were included (Number of Roles and Year Experience). This result was unexpected given that variability in interpersonal communication has been shown to predict situational awareness (Buchler et al., 2016a) and overall team performance (Monge and Contractor, 2001; Henshel et al., 2016) in other contexts. This exact measure uniquely predicted Scenario Injects performance in the 2016 MACCDC (Buchler et al., 2018). From this we can infer that either improved Collaboration covary with increased Experience and role specialization or that our measure was not able to capture the unique aspects of teamwork.

Finally, years of Experience was a uniquely positive predictor for the Maintaining Services and Incident Response scores. It was also positively associated with Scenario Injects scores but not as a unique predictor. These results support Dodge et al. (2007)'s earlier findings that seniors are tougher targets than freshman in capture-the-flag cyber competitions. Experience-related change is a hallmark of team development and skill acquisition. Our results support theories of team development and highlight functional role specialization as a key potential indicator of developmental stage.

### 6.4. Model comparison across subsequent competitions (MACCDC 2016-2017)

**Figure 9:**
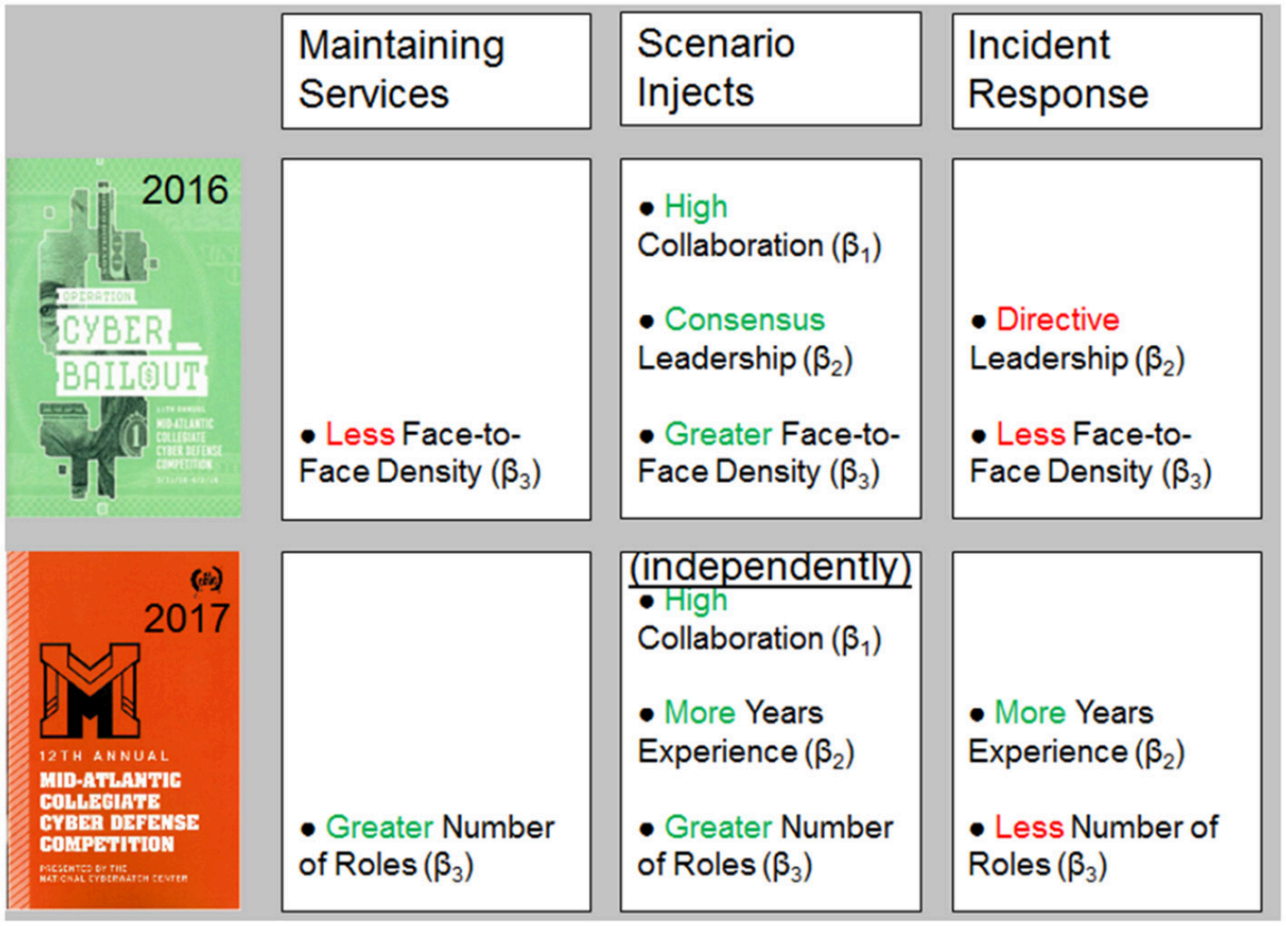
Comparison of Bayesian Multiple Linear Regression Models predictors across the MACCDC 2016 **(Top)** and MACCDC 2017 **(Bottom)** events for the scored performance dimensions of **(Left)** Maintaining Services, **(Middle)** Scenario Injects, **(Right)** Incident Response.

To facilitate model comparison, similar data analysis was conducted to the preceding event, the MACCDC 2016 (Buchler et al., 2018). Both analyses conducted at the 2016 and 2017 MACCDC events examine potential predictors or determinants of effective cyber teaming using Bayesian analytical methodologies with scored performance as a outcome measure. The subsequent MACCDC events are professionally consistent, with the same three scoring dimensions: (a) Maintaining Services, (b) Scenario Injects, and (c) Incident Response. Every year a different scenario is featured. The 2016 MACCDC *Operation Cyber Bailout* scenario involving cyber attacks on a mid size financial institution, whereas the 2017 MACCDC featured the *Man vs. Machine* scenario, described earlier. A model comparison of significant predictors for the MACCDC 2016 and the current MACCDC 2017 events is shown in Figure 9 for each of the three soring dimensions. Our current MACCDC 2017 *Man vs. Machine* results are summarized across the lower row as significant predictors of performance.

Sociometrics were used in the 2016 event as a measure of team structure (Face-to-Face Density) derived from wearable sensors (Sociometric Badges) that detected interpersonal interactions among team-members. Reviewing the 2016 MACCDC results (top row) in comparison to our current findings, teams structured with less Face-to-Face Density tended to score higher for Maintaining Services. This finding was consistent with Tuckman's (1965) stage model of team formation as low performing teams exhibited greater Face-to-Face Density of interactions, whereas high performing teams had established normative work routines and may have functional role specialization that would limit the need for face-to-face interactions. Team members were compartmentalized and knew what needed to be done to accomplish the various tasks in the scored competition. Indeed, our current model found that teams with a greater Number of Roles tended to have higher scores for Maintaining Services, supporting our earlier hypothesis that teams engage in functional role specialization.

For Scenario Injects, teams receive high-priority tasks that need to be completed quickly against the clock. All three derived measures were predictors of scored task performance: rich collaboration, consensus (non-directive) leadership style, and greater face-to-face density. This suggests that a high degree of coordination was necessary to respond effectively. Our findings replicated this result that rich Collaboration was important in individual regressions, but not overall; this suggests that greater Number of Roles and more Years Experience are inter-related with a high degree of Collaboration.

For Incident Response, our previous results found that lower Face-to-Face Density also emerged as a strong predictor of scored performance as well as a directive Leadership style. Thus, to perform well on Incident Response, teams did well to focus on the task at hand by limiting face-to-face interactions and adopt more of a directive Leadership style in analyzing and coordinating all of the information required in writing up a report of each cyber incident and reporting it to authorities. This is consistent with present finding that high Incident Response scores were associated with more Years Experience (professionalism) and with fewer Number of Roles focused on system-level analyses (task at hand).

The key goal in our earlier analysis was to extend our approach to address team development and maturity level to include overall amount of experience working as a team as well as team composition. The present work is consistent with our previous findings and fulfills that goal.

### 6.5. Effective teams

Team effectiveness refers to the work-directed capacity of a team to accomplish the defined goals and objectives of their organization (Hackman and Hackman, 2002). In our case, team effectiveness was clearly defined as an outcome variable by the score of our cyber teams in the competition. Group dynamics evolve over time, and for highly proficient teams, there is general agreement in the broad research literature examining real work-directed teams ranging from medical teams, to air traffic control, military squads, and intelligence analysts that as individuals become accustomed to performing tasks together they develop shared team cognition —defined theoretically as the collective knowledge and experience of the team, encapsulated as reciprocal mental models, that allows them to anticipate one another, coordinate, and ultimately achieve effective and efficient workflows (Cannon-Bowers et al., 1993). The Shared Mental Models theory suggests that team performance depends on the degree to which knowledge and understanding of the situation and task-level workflows are similarly understood across team-members (Cannon-Bowers and Salas, 2001). A limitation of the mental model theory is that it does not explain the role that communications plays and how the members of effective teams talk to one another (Rajivan and Cooke, 2017). More recent adaptations, such as the Interactive Team Cognition approach focuses on observable communications and intermediary collaborative work-products as providing key insights into team dynamics (Cooke et al., 2013). Team members develop transactive memory (Wegner, 1987), the distributed knowledge that guides inter-team communications and collaborations, by efficiently remembering who does what on the team. Our findings fit well with these theories of team cognition and we extend the model to address how the depth and breadth of functional skills on the team contribute to organizational agility and team effectiveness.

We propose a *functional team cognition* framework that we based on team members' knowledge of the skills and proficiency-level of their teammates in relation to work processes. In the context of a competition or other high-demand work environments, we posit that functional team cognition may enable organizational agility in the effective allocation of team members work capabilities to the presenting task demands. This allows them to self-organize and achieve team synchronization as a case of distributed decision making (Rasmussen et al., 1991; Hutchins et al., 2001; for a model, see Dekker, 2011) especially during peak work-demands and time-stressed or resource-constrained situations. We base our assertion on our results that high-performing teams in the cyber competition were composed of experienced members with multiple overlapping roles, demonstrating both skill breadth and depth. These results are consistent with Gersick and Davis-Sacks (1990)'s argument that product development teams were successful in part because “members had a relatively good idea about who had what expertise, and they knew they were highly interdependent in their effort to reach a shared and consequential objective” (p. 148). Furthermore, teams with depth and breadth are better able to adapt to the uncertain heterogeneous factors that can influence success (p. 153). In time-stressed or resource-constrained situations, we argue that skill depth and breadth also enables organizational agility as multiple team members can self-organize to address current challenges and avoid bottlenecks in teamwork and resource constraints that are highly-dependent upon a particular skill-set or utility. In this case, team-members can adapt to situational demands and take on multiple tasks concurrently and flexibly to achieve desirable outcomes.

Functional task allocation can also be dynamically managed by a team leader (for a meta-analysis establishing empowerment behaviors, Burke et al., 2006). For instance, a team leader can more successfully direct team members to respond to a given scenario event or deliver a full incident report work product if: (1) their team has multiple members with relevant experience (i.e., intrusion detection system and netflow), and (2) the team leader has an accurate mental model of the skill competencies of various team members. In future work, we plan to test our *functional team cognition* framework by using consensus analysis to operationally define a metric of assessment and furthermore, to evaluate whether teams that have a high-degree of shared knowledge of team members' skill profiles perform better than teams that do not. The role of leadership in developing functional team cognition is another area of interest. Based on our results, we hypothesize that directive leadership may be able to compensate in cases where functional team cognition is low among the team members, evident with poor reported consensus among team members about each others' skills. In this case, a leader with an accurate mental model of the skill competencies of the various team members could adopt a directive leadership style to assign and coordinate team members to address current work demands.

### 6.6. Conclusion

Our results are in line with theories of team formation/maturation (Tuckman, 1965) and extend our previous work (Buchler et al., 2018) by demonstrating that more competitive experience and team functional role specialization are strongly associated with successful performance in cyber-defense competitions. This work highlights the need to evaluate team level factors and the team members' characteristics when predicting whether a mission will be successful. It also demonstrates that the benefits of a particular team-level skill composition are dependent on the type of task the team will need to complete. A key objective of future work is to evaluate whether these results replicate at other events in laboratory based simulation studies and to determine the team skill profiles that are ideal for other cyber defense tasks.

## Author contributions

NB contributed to the ideas, design, execution of the study as well as the analyses of results and write up of the manuscript. CL contributed to the analyses of results and write up of the manuscript. BH contributed to the design and execution of the study as well as the write up of the manuscript. PR contributed to the analyses of results. LM contributed to the analyses of results. LL contributed to the design and execution of the study.

### Conflict of interest statement

The authors declare that the research was conducted in the absence of any commercial or financial relationships that could be construed as a potential conflict of interest.

## Footnotes

^1^www.nationalcyberwatch.org - a consortium of higher education institutions, public and private schools, businesses, and government agencies focused on collaborative efforts to advance cybersecurity education and strengthen the national cybersecurity workforce.

## Supplementary material

Figure S1 **(A)** Posterior distributions of regression parameters for Model 2 predicting Scenario Inject score with β*1* (Communication & Collaboration Factor), β*2* (Years Experience), and β*3* (Number of Roles) as predictors of team performance. Each of these predictors were significant independently, noting common variance. **(B)** Posterior distributions of difference among parameters indicate inter-related predictive quality of β*1*, β*2* and β*3* to Scenario Inject scored performance.
