# Supplementary figures and images for: Cyber Teaming and Role Specialization in a Cyber Security Defense Competition
Source: Front Psychol. 2018 Nov 19;9:2133. doi: 10.3389/fpsyg.2018.02133 (PMC6252333; doi:10.3389/fpsyg.2018.02133)

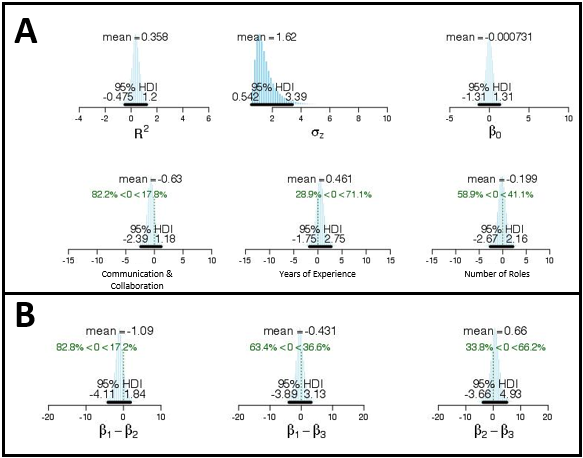

Supplement: Figure S1 — (A) Posterior distributions of regression parameters for Model 2 predicting Scenario Inject score with β1 (Communication & Collaboration Factor), β2 (Years Experience), and β3 (Number of Roles) as predictors of team performance. Each of these predictors were significant independently, noting common variance. (B) Posterior distributions of difference among parameters indicate inter-related predictive quality of β1, β2 and β3 to Scenario Inject scored performance. [file Image_1.png]
